# Risk factors for smoking in adolescence: evidence from a cross-sectional survey in Switzerland

**DOI:** 10.1186/s12889-024-18695-4

**Published:** 2024-04-25

**Authors:** Szilvia Altwicker-Hámori, Kurt Alexander Ackermann, Pia Furchheim, Julia Dratva, Dominique Truninger, Steffen Müller, Frank Wieber

**Affiliations:** 1 Winterthur Institute of Health Economics, School of Management and Law, ZHAW Zürich University of Applied Sciences, Winterthur, Switzerland; 2 Center for Behavioral Insights & Pricing, School of Management and Law, ZHAW Zürich University of Applied Sciences, Winterthur, Switzerland; 3 Institute of Public Health, School of Health Sciences, ZHAW Zürich University of Applied Sciences, Winterthur, Switzerland; 4 Medical Faculty, University of Basel, Basel, Switzerland; 5 Department of Psychology, University of Konstanz, Konstanz, Germany

**Keywords:** Adolescence, Smoking behaviour, Smoking prevention, Switzerland

## Abstract

**Background:**

Cigarette smoking during adolescence is a major public health concern with far-reaching health implications. Adolescents who smoke are at an increased risk of developing long-term health problems and are more likely to continue smoking into adulthood. Therefore, it is vital to identify and understand the risk factors that contribute to adolescent smoking – which in turn facilitate the development of targeted prevention and intervention programs.

**Methods:**

Data was drawn from a cross-sectional survey conducted between October and December 2021, encompassing adolescents of adolescents aged 14 to 19 residing in Switzerland (*n* = 2,683). Multiple logistic regression analysis was employed to explore which demographic, household, behavioural and psychographic factors are associated with current smoking status.

**Results:**

The regression results showed higher odds of smoking for female respondents (OR 1.39; *p*-value 0.007); older adolescents (OR 1.30; *p*-value < 0.001); those living in the French-speaking part of Switzerland (OR 1.39; *p*-value 0.021), in suburban areas (OR 1.35; *p*-value 0.023) and with a smoker in the same household (OR 2.41; *p*-value < 0.001); adolescents consuming alcohol (OR 4.10; *p*-value < 0.001), cannabis products (OR 6.72; *p*-value < 0.001) and hookah (OR 5.07; *p*-value < 0.001) at least once a month; respondents not engaging in sports (OR 1.90; *p*-value < 0.001) or music (OR 1.42; *p*-value 0.031) as top five leisure activities and those experiencing high stress levels at home (OR 1.74; *p*-value < 0.001). Adolescents with high scores in health awareness (OR 0.33; *p*-value < 0.001), on the relational self-esteem scale (OR 0.78; *p*-value 0.054) and on the general well-being scale (OR 0.52; *p*-value 0.022) were less likely to smoke than their counterparts with lower scores. High risk-seeking was associated with higher odds of smoking (OR 2.15; *p*-value < 0.001).

**Conclusions:**

Our results suggest the importance of a comprehensive approach at both individual and institutional levels to reduce smoking rates in adolescents. More specifically, a holistic strategy that encompasses adolescents, families, schools and policymakers ranging from strengthening adolescents’ self-esteem, smoking cessation support for parents, to increasing engagement in musical and physical activities, and enhancing health awareness in the school curriculum.

## Background

Cigarette smoking during adolescence is a critical global public health concern with far-reaching health implications. Young people require fewer cigarettes and less time compared to adults to develop a nicotine addiction [1]. Adolescents who smoke are at an increased risk of developing long-term health problems and are more likely to continue smoking into adulthood, further exacerbating the burden of tobacco-related diseases [1–4].

Adolescents in Switzerland are particularly vulnerable. Despite various measures over the past decades, such as the national law to protect people from passive smoking which took effect in May 2010, Swiss legislation on protection against tobacco, nicotine, and their consequences is considered very weak compared to Europe and the rest of the world [5]. Notably, Switzerland is among the few countries that have not ratified the WHO’s Framework Convention on Tobacco Control [5]. Consequently, the Swiss association for tobacco prioritizes the protection of young people [5]. This is imminent as, according to the 2017 Swiss Health Survey (SHS), 32% of respondents aged 15 to 24 reported current smoking [6]. Given that early exposure to nicotine increases the severity of dependence later in life [7], it is particularly concerning that in 2018 in Switzerland, 8.7% of 15-year-olds smoked at least once a week [8]. It is also concerning that, according to the 2017 SHS, only 44% of individuals who started smoking managed to quit; despite the fact that 61% of smokers express a desire to quit smoking [9].

It is therefore vital to identify the risk factors that contribute to adolescent smoking in Switzerland. By identifying these factors, the current study may inform evidence-based policymaking and targeted intervention programs aimed at reducing smoking rates in adolescents.

We selected variables identified in existing studies as significant risk factors for adolescent smoking in our statistical analysis. This allows us to contextualize our findings within the broader literature and explore the unique interplay of these factors within the Swiss adolescent population. These factors include demographic factors (such as age [10, 11] and region of residence [12–14]), household characteristics (e.g., parental smoking [10, 11, 15–19]), behavioural factors (such as substance use [10, 20, 21] and leisure activity [22–25]) as well as psychographic factors (including risk taking behaviour [22, 26] and self-esteem [10, 19, 26–28]). These potential risk factors were collected in a cross-sectional survey conducted in 2021 as part of the REACH Project. The survey targeted adolescents aged 14 to 19, a particularly vulnerable period for smoking initiation [29]. This survey thus serves as an excellent data source for our study.

## Methods

### Data and sample selection

Data for the statistical analysis was drawn from a cross-sectional survey of adolescents living in Switzerland, conducted as part of the REACH Project [29]. Adolescents aged 14 to 19 were recruited between October and December of 2021. The primary recruitment channels included public secondary schools in eight cantons (Aargau, Bern, Geneva, Jura, Lucerne, Thurgau, Ticino and Vaud), various youth organizations and clubs throughout Switzerland as well as social media platforms (including Instagram and Facebook). To encourage participation, the approximately 15-minute online-questionnaire was made available in three of the four national languages (German, French and Italian) and participants had the opportunity to win prizes. Further information on the survey can be found at the Swiss association for tobacco control website [29].

The initial dataset included 2,732 respondents, with missing values identified for only two variables, i.e., migration background (*n*_*1*_ = 33) and language region of residence (*n*_*2*_ = 16). Given that these missing values accounted for less than 1% of the sample for each variable, we excluded them for the analysis. Our final sample comprised 2,683 individuals.

### Outcome variable

The outcome variable was a binary variable equal to one for current smokers and zero for current non-smokers (i.e., those who answered with “yes” and “no” to the question “Do you smoke?”, respectively). The latter group included ex-smokers and never-smokers.

#### Independent variables

The five demographic variables included in our analysis encompassed gender (“male, “female”, “other”); age (14 to 19); migration background (“both parents born in Switzerland”, “one parent born in Switzerland”, “both parents born outside Switzerland”); language region of residence (“German”, “French”, “Italian”) and urbanicity of the place of residence (“rural”, “suburban, “urban”).

We included two household variables in the analysis: first, an indicator variable for smoker living in the same household; second, three categories capturing the financial situation of the household: (1) “We are in a good financial situation and can afford many things, but we still have to be mindful of our money.” or better; (2) “We have enough, and it’s sufficient to treat ourselves occasionally.” or worse; and (3) “I do not know.”. These latter three categories were aggregated from the eight response options provided in the survey: (1) “We can’t make ends meet on our own and need outside help (e.g., daily allowances, social assistance, subsidies etc.).”; (2) “We just barely make ends meet, but we have to turn over every cent.”; (3) “We get by, but we can’t afford to do anything great.”; (4) “We have enough, and it’s sufficient to treat ourselves occasionally.”; (5) “We are in a good financial situation and can afford a lot of things, but we still have to be mindful about money.”; (6) “We are in a very good financial situation and can practically afford anything we want.”; (7) ”We can afford anything we want – money is not an issue.”; (8) “I don’t know.”

The six behavioural variables used in the analysis included indicator variables for the frequency of alcohol consumption, the frequency cannabis/marijuana/hashish consumption, the frequency of hookah consumption, the frequency of social media consumption (based on the James-Study [30] as well as engagement in sports and musical activities. To construct the latter variables “Top five leisure activity: Sports” and “Top five leisure activity: Music” the responses from the following question were used: “What do you usually do in your free time? Please choose from this list the activities you do most frequently during the week. You can name up to five leisure activities.”. For the former variable, the response options, “participating in organised sports (e.g., playing football or volleyball regularly in a club)” and “engaging in sports individually (e.g., cycling or running alone)” were combined; for the latter variable the response options “engaging in organised musical activities (e.g., in music school, choir or orchestra)” and “making music alone for myself” were aggregated.

Eight psychographic binary variables were generated for the statistical analysis to indicate levels of stress at home/with family members, sensation seeking [31], health awareness, risk-seeking [32], affiliation motivation [33], openness [34], relational self-esteem [35]and general well-being [36]. While the subsequent text describes these eight variables and their respective values as presented in the survey, Table 1 presents the corresponding dichotomous variables. In the survey, to assess current stress levels at home, participants were asked to rate the question “To what extent do you currently feel stressed or under pressure at home/from your parents?” using a six-point scale, with 1 indicating “not stressed at all” and 6 indicating “very heavily stressed”. To assess sensation seeking tendencies, participants were requested to rate their agreement with the statement “I want to experience as much as possible. There is nothing worse than boredom.” on a six-point scale, ranging from 1 (strongly disagree) to 6 (strongly agree). To measure levels of health awareness, participants were instructed to evaluate their lifestyle (i.e., “How would you describe your lifestyle?”) using a six-point scale, ranging from 1 (not at all health-aware) to 6 (very health-aware). To evaluate risk-seeking tendencies, affiliation and openness tendencies, participants were asked to rate their agreement with the statement “I am generally a risk-taking person.“, “I have a strong need to belong.” and “I am open to new experiences and enjoy trying things that I don’t yet know if I like.” on a six-point scale, ranging from 1 (strongly disagree) to 6 (strongly agree), respectively.

To evaluate relational self-esteem, the following six of the seven items of a validated relational self-esteem scale [35] were included in the survey: (1) “I am a worthy member of my circle of friends.”, (2) “Overall, my circle of friends is considered good by others.”, (3) “I think my family is proud of me.”, (4) “In general, I am happy to be a member of my circle of friends.”, (5) “I can help my friends a lot.” and (6) “I feel that I have much to offer to my family.” Participants were requested to rate each item on a six-point scale, ranging from 1 (completely disagree) to 6 (completely agree). To assess overall general well-being, the WHO-5 Well-Being Index [36] with slight modifications was incorporated in the survey: (1) “My daily life is filled with things that interest me.”, (2) “I feel calm and relaxed.”, (3) “I wake up feeling fresh and rested.”, (4) “I feel active and vigorous.” and (5) “I feel cheerful and in good spirits.”. Participants were instructed to rate each item on a six-point scale, where 1 indicated “never” and 6 indicated “always”.

Table 1 presents the independent variables used in the analysis and their corresponding values.

**Table 1:** Description of independent variables

Variable	Value
*Demographic*	
Gender	0 = male 1 = female 2 = other
Age	14–19
Migration background	0 = both parents born in Switzerland 1 = one parent born in Switzerland 2 = both parents born outside Switzerland
Language region of residence	0 = German 1 = French 2 = Italian
Urbanicity of place of residence	0 = rural 1 = suburban 2 = urban
*Household*	
Financial situation of household	0=“We are in a good financial situation and can afford many things, but we still have to be mindful of our money.” or better 1=“We have enough, and it’s sufficient to treat ourselves occasionally.” or worse 2=“I do not know.”
Smoker living in the same household	0 = no 1 = yes
*Behavioural*	
Frequency of alcohol consumption	0 = 3 or less 1 = at least 4*
Frequency of cannabis/marijuana/hashish consumption	0 = 3 or less 1 = at least 4*
Frequency of hookah consumption	0 = 3 or less 1 = at least 4*
Frequency of social media consumption	0 = daily or less often 1 = several times a day**
Top five leisure activity: Sports	0 = yes 1 = no
Top five leisure activity: Music	0 = yes 1 = no
*Psychographic*	
Stress level at home/with family members	0 = 3 or less 1 = at least 4***
Sensation seeking	0 = 4 or less 1 = at least 5***
Health awareness	0 = 4 or less 1 = at least 5***
Risk-seeking	0 = 4 or less 1 = at least 5***
Affiliation motivation	0 = 4 or less 1 = at least 5***
Openness	0 = 4 or less 1 = at least 5***
Relational self-esteem	0 = 4 or less 1 = at least 5***
General well-being	0 = 4 or less 1 = at least 5***

Category 0 corresponds to the reference category. *Response options: 1 = no, never; 2 = no, but have already tried before; 3 = yes, but less than once a month; 4 = yes, at least once a month; 5 = yes, at least once a week; 6 = yes, daily. **Response options: 1 = never, 2 = less than once a week, 3 = several times a month, 4 = several times a week, 5 = daily, 6 = several times a day. ***Response options described in text above (“Independent Variables”)

### Analysis

Multiple logistic regression analysis was employed to explore the associations between current smoking status and demographic, household, behavioural and psychographic variables (see Table 1). Regression results are presented as odds ratios (OR) with *p*-values and 95% confidence intervals (95% CI). A *p*-value of 0.05 was regarded as statistically significant in all analyses. All statistical analyses were conducted using Stata 17.

## Results

### Descriptive statistics

Sample descriptive statistics are provided in Table 2. One-quarter of the participants were smokers. In terms of gender identification, about 60% of the participants identified as “female”, 38% as “male” and 2% as “other”. The mean age of the participants was 16.1 years (standard deviation 1.62). Three-quarters of the participants had at least one parent who was born in Switzerland. Roughly 60% of the participants resided in the German-speaking part of Switzerland, 26% in the French-speaking part and 14% in the Italian-speaking part. Nearly half of the sample (47%) lived in rural areas.

In terms of household characteristics, about 52% of the participants described their financial situation as “We are in a good financial situation and can afford many things, but we still have to be mindful of our money.” or better and 37% lived with a smoker in the same household.

Approximately 36% of the respondents reported consuming alcohol at least once a month; the corresponding figures for cannabis/marijuana/hashish and hookah consumption were 9% and 3%, respectively. The majority of the respondents (56%) used social media on a daily basis. Approximately half of the sample (49%) indicated organised or individual sports as their top five leisure activity; the respective figure for engaging in organised or individual musical activities was only 17%.

Around 24% of the respondents reported experiencing high stress levels at home or with family members (i.e., at least four on a six-point-rating scale). Regarding the other psychographic variables, 50% of the sample scored high (i.e., at least five a six-point-rating scale) in sensation seeking, 13% in health awareness, 28% in risk seeking, around 36% in affiliation motivation, 45% in openness, 35% on the relational self-esteem scale and only 10% on the general well-being scale.

**Table 2:** Descriptive statistics (*n* = 2,683)

Variable	Percent
*Outcome*	
Non-smoker Smoker	74.7 25.3
*Demographic*	
Gender	
Male Female Other	38.1 60.2 1.7
Age	16.1 (1.62) [14, 19]
Migration background	
Both parents born in Switzerland One parent born in Switzerland Both parents born outside Switzerland	53.3 21.5 25.2
Language region of residence	
German French Italian	59.7 25.9 14.4
Urbanicity of place of residence	
Rural Suburban Urban	46.9 31.6 21.5
*Household*	
*Financial situation*	
“We are in a good financial situation and can afford many things, but we still have to be mindful of our money.” or better “We have enough, and it’s sufficient to treat ourselves occasionally.” or worse “I do not know”	51.9 37.2 10.9
Smoker living in the same household	37.0
*Behavioural*	
Frequency of alcohol consumption: at least 4*	35.9
Frequency of cannabis/marijuana/hashish consumption: at least 4*	9.1
Frequency of hookah consumption: at least 4*	3.2
Frequency of social media consumption: several times a day**	56.1
Top five leisure activity: Sports: no	50.9
Top five leisure activity: Music: no	82.9
*Psychographic*	
Stress level at home/with family members: at least 4***	23.9
Sensation seeking: at least 5***	49.5
Health awareness: at least 5***	13.0
Risk-seeking: at least 5***	28.2
Affiliation motivation: at least 5***	35.9
Openness: at least 5***	44.5
Relational self-esteem: at least 5***	35.0
General well-being: at least 5***	9.9

*Response options: 1 = no, never; 2 = no, but have already tried before; 3 = yes, but less than once a month; 4 = yes, at least once a month; 5 = yes, at least once a week; 6 = yes, daily. **Response options: 1 = never, 2 = less than once a week, 3 = several times a month, 4 = several times a week, 5 = daily, 6 = several times a day. ***Response options described in text above (“Independent Variables”). Standard deviation in brackets; range in squared brackets

### Regression analysis

The regression results are presented in Table 3. Female respondents were more likely to be smokers than their male counterparts (OR 1.39; 95% CI 1.09–1.77; *p*-value 0.007). Older adolescents had higher odds of smoking than their younger counterparts (OR 1.30; 95% CI 1.21–1.41; *p*-value < 0.001). Adolescents living in the French-speaking part of Switzerland were more likely to smoke than their counterparts living in the German-speaking part (OR 1.39; 95% CI 1.05–1.84; *p*-value 0.021). Suburban residents were more likely to be smokers than comparable urban residents (OR 1.35; 95% CI 1.04–1.76; *p*-value 0.023).

Adolescents who reported to be living with a smoker in the same household had higher odds of smoking than comparable adolescents living without a smoker (OR 2.41; 95% CI 1.92–3.02; *p*-value < 0.001).

Frequent (i.e., at least once a month) alcohol consumption (OR 4.10; 95% CI 3.20–5.25; *p*-value < 0.001), cannabis/marijuana/hashish consumption (OR 6.72; 95% CI 4.68–9.67; *p*-value < 0.001) and hookah consumption (OR 5.07; 95% CI 2.91–8.85; *p*-value < 0.001) were associated with higher odds of smoking. The lack of physical (i.e., organised or individual sports) and musical activity was also associated with higher odds of smoking (OR 1.90; 95% CI 1.50–2.39; *p*-value < 0.001 and OR 1.42; 95% CI 1.03–1.95; *p*-value 0.031), respectively.

Adolescents experiencing high stress levels at home were more likely to be smokers (OR 1.74; 95% CI 1.35–2.24; *p*-value < 0.001). Adolescents with high scores in health awareness (OR 0.33; 95% CI 0.19–0.56; *p*-value < 0.001), on the relational self-esteem scale (OR 0.78; 95% CI 0.60–1.00; *p*-value 0.054) and on the general well-being scale (OR 0.52; 95% CI 0.29–0.91; *p*-value 0.022) were less likely to smoke than their counterparts with lower scores. On the other hand, high risk-seeking was associated with higher odds of smoking (OR 2.15; 95% CI 1.65–2.80; *p*-value < 0.001).

No statistically significant association was found between current smoking status and migration background, household’s financial situation, social media consumption, sensation seeking, affiliation motivation and openness.

**Table 3:** Multiple logistic regression model with current smoking as outcome (*n* = 2,683)

	OR	95% CI	*p*-value
Gender: male	1		
Gender: female	1.39	(1.09–1.77)	0.007
Gender: other	1.50	(0.66–3.40)	0.328
Age	1.30	(1.21–1.41)	< 0.001
Migration background: Both parents born in Switzerland	1		
Migration background: one parent born in Switzerland	1.17	(0.88–1.55)	0.279
Migration background: both parents born in Switzerland	1.28	(0.96–1.69)	0.090
Language region of residence: German	1		
Language region of residence: French	1.39	(1.05–1.84)	0.021
Language region of residence: Italian	0.95	(0.70–1.30)	0.743
Urbanicity of place of residence: rural	1		
Urbanicity of place of residence: suburban	1.35	(1.04–1.76)	0.023
Urbanicity of place of residence: urban	1.27	(0.95–1.69)	0.109
Financial situation: ““We are in a good financial situation and can afford many things, but we still have to be mindful of our money.” or better	1		
Financial situation: “We have enough, and it’s sufficient to treat ourselves occasionally.” or worse	1.11	(0.87–1.41)	0.406
Financial situation: “I do not know.”	1.07	(0.71–1.61)	0.738
Smoker living in the same household: no	1		
Smoker living in the same household: yes	2.41	(1.92–3.02)	< 0.001
Frequency of alcohol consumption: 3 or less	1		
Frequency of alcohol consumption: at least 4	4.10	(3.20–5.25)	< 0.001
Frequency of cannabis/marijuana/hashish consumption: 3 or less	1		
Frequency of cannabis/marijuana/hashish consumption: at least 4	6.72	(4.68–9.67)	< 0.001
Frequency of hookah consumption: 3 or less	1		
Frequency of hookah consumption: at least 4	5.07	(2.91–8.85)	< 0.001
Frequency of social media consumption: daily or less often	1		
Frequency of social media consumption: several times a day	1.05	(0.83–1.31)	0.703
Top five leisure activities: Sports: yes	1		
Top five leisure activities: Sports: no	1.90	(1.50–2.39)	< 0.001
Top five leisure activities: Music: yes	1		
Top five leisure activity: Music: no	1.42	(1.03–1.95)	0.031
Stress level at home/with family members: 3 or less	1		
Stress level at home/with family members: at least 4	1.74	(1.35–2.24)	< 0.001
Sensation seeking: 4 or less	1		
Sensation seeking: at least 5	1.14	(0.89–1.46)	0.309
Health awareness: 4 or less	1		
Health awareness: at least 5	0.33	(0.19–0.56)	< 0.001
Risk-seeking: 4 or less	1		
Risk-seeking: at least 5	2.15	(1.65–2.80)	< 0.001
Affiliation motivation: 4 or less	1		
Affiliation motivation: at least 5	0.92	(0.73–1.16)	0.495
Openness: 4 or less	1		
Openness: at least 5	1.00	(0.78–1.28)	1.000
Relational self-esteem: 4 or less	1		
Relational self-esteem: at least 5	0.78	(0.60–1.00)	0.054
General well-being: 4 or less	1		
General well-being: at least 5	0.52	(0.29–0.91)	0.022

OR: odds ratio. CI: confidence interval. See Table 1 and Section “Independent variables” for a description of the variables and their values

As a robustness check, the analysis was conducted excluding ex-smokers (*n*_*3*_ = 74) from the sample. The coefficient estimates obtained in this subsample analysis were consistent with those of the full sample (results available upon request).

## Discussion

### Main findings

This study examined the risk factors associated with smoking behaviour among 14 to 19-year-old adolescents living in Switzerland using a cross-sectional survey. The findings revealed higher odds of smoking for female respondents; older adolescents; those living in the French-speaking part of Switzerland, in suburban areas and with a smoker in the same household. Adolescents consuming alcohol, hookah and cannabis products at least once a month and not engaging in sports or musical activities as their top five leisure activities were also more likely to smoke. Those experiencing high stress levels at home, exhibiting high risk-seeking behaviour, low health awareness, low relational self-esteem and low general well-being also showed higher odds of smoking.

Parental smoking is a well-documented risk factor for adolescent smoking [10, 11, 15–19]. For example, a multigenerational study in the US revealed that adolescents with parents who were nicotine-dependent smokers at baseline were more likely to be early regular smokers and early experimenters with each additional year of previous exposure to parental smoking [16]. In our study, we cannot disentangle the role of environmental and genetic factors in adolescent smoking – albeit a highly relevant topic [37, 38]. Nevertheless, our results indicate the need for interventions to help nicotine-dependent parents quit smoking early in their children’s lifetime.

Our results regarding behavioural factors are in line with international research findings. A systematic review analysing the co-use of tobacco and marijuana based on 163 English language articles found that the use of one substance increases the likelihood of concurrent use of the other substance [20]. Another systematic review examining cigarette smoking trajectories in adolescents revealed that both alcohol and cannabis use are associated with trajectory membership [39]. Moreover, Orlando and colleagues find evidence indicating that whereas it is common during adolescence to drink but not smoke, it is very rare to smoke and not drink [21]. Furthermore, alcohol and drug abuse have been shown to be associated with smoking initiation in adolescent girls [22].

Cigarette smoking may be viewed as a coping mechanism for dealing with anxiety and other psychological distress triggered by environmental stressors [22]. More effective and healthy options to cope with anxiety and stress comprise participating in organised sports [22, 23] and engaging in musical activities [24, 25]. For instance, a study by Cabane and colleagues found that engaging in both activities, music and sports, reduces smoking by about 10%-points compared to engaging in just one activity [24]. Overall, our findings point to the importance of integrating comprehensive programs at the school level from an early age. It appears crucial to enhance (1) health awareness by addressing the dangers of smoking, for example, by integrating substance abuse education into the curriculum as well as (2) engagement in musical and physical activities. The latter aligns with the WHO’s recommendation for children and adolescents aged five to 17 years, which suggests an average 60 min per day of moderate-to-vigorous-intensity exercise during the week [40].

While risk-taking behaviour has been consistently shown to be associated with smoking initiation [22], the association between self-esteem and substance use, including cigarette smoking, often varies depending on how self-esteem is measured [26]. Our study specifically focused on relational self-esteem, examining how adolescents perceive themselves within their circle of friends and family. Our findings suggest the relevance of interventions targeting both school and home environments. School-level initiatives, such as peer mentoring programs designed to strengthen social skills, and parental guidance interventions aimed at equipping parents with the skills to create a supportive atmosphere, may contribute to more positive self-perceptions within friend and family circles [26], ultimately helping adolescents to cope with challenges and resist cigarette smoking.

Finally, our study aligns with the trend observed between French and German-speaking regions in Switzerland. According to the 2017 SHS, the rate of smoking was statistically significantly higher in the Italian-speaking Switzerland than in the German-speaking regions, with the French-speaking regions falling in between [14]. Similarly, based on the 2016 Continuous Rolling Survey of Addictive Behaviours and Related Risks (CoRoIAR), the smoking prevalence was lower in the German-speaking region compared to the French- and Italian-speaking regions among participants aged 14 to 25 [13].

### Methodological considerations

The main strengths of our study stem from the data source, which was collected in the REACH project. These strengths include (1) the use of population-based data, (2) the inclusion of a relatively large sample of survey respondents ensuring meaningful statistical analysis, (3) the high data quality in terms of missing responses (i.e., less than 2% in just two variables), (4) the mitigation of potential recall bias through the focus on present behaviours and feelings in the survey and (5) the inclusion of a large percentage of socio-economically less well-off adolescents – a population often challenging to reach.

Nevertheless, our study has its limitations. Most importantly, our study cannot establish causation only associations – a limitation that calls for caution when informing policymaking. Second, considering the evidence that in Switzerland, both smoking prevalence and intensity suffer from underreporting [41], underestimation of smoking cannot be ruled out completely. However, underreporting in smoking status is alleviated by the anonymous data collection. Third, the overrepresentation of participants from the Italian-speaking part of Switzerland – were smoking prevalence has been found to be higher than in the German-speaking regions among individuals aged 14 to 25 [13] – may inflate smoking prevalence in our study. Moreover, the higher participation probability of Italian-speaking youth may introduce bias into our regression models, even after controlling for language-speaking regions. For instance, if Italian-speaking adolescents have unique cultural, socio-economic or environmental characteristics influencing their smoking behaviour differently than other groups, this overrepresentation could lead to misleading conclusions about risk factors and effective interventions at the national level. Therefore, supplementing our findings with representative data sources and conducting separate analyses in language regions using large datasets are crucial for validation in future research and informing policy decisions. Finally, because of the cross-sectional design, we cannot observe changes over time. Future research should address these limitations, for instance, by employing causal designs and recent longitudinal data from different sources.

Two additional aspects merit future research. First, considering recent legal developments – such as the implementation of the strictest nationwide smoking ban in the canton of Geneva starting 1st of June 2023 [42], and the Swiss people’s approval of the “Children Without Tobacco” initiative in February 2022 (with ongoing legislation process [43]) – regional differences, also at a more disaggregated level, should be re-addressed in future research. To this end, our findings provide a suitable baseline. Second, given the study’s focus on relational self-esteem within the circle of friends and family, the integration of a multi-dimensional self-esteem scale may prove to be beneficial. Third, our study is based on data from the COVID-19 pandemic period. It is important to note that Switzerland did not implement a lockdown during the three-month study period – a measure which according to international evidence may affect smoking behaviour [44]. Nevertheless, analysing the smoking behaviour for this age group based on post-COVID-19 data would be valuable; especially in light of the international evidence that the impact of COVID-19 pandemic on smoking consumption is unclear and complex [45]. Finally, future analyses using more disaggregated categories, based on a large dataset, should yield additional in-depth findings.

## Conclusions

Our results suggest the importance of a comprehensive approach at both individual and institutional levels to reduce smoking rates in adolescents. More specifically, a holistic strategy that encompasses adolescents, families, schools and policymakers ranging from strengthening adolescents’ self-esteem, smoking cessation support for parents, to increasing engagement in musical and physical activities, and enhancing health awareness in the school curriculum. Furthermore, our findings provide a suitable baseline for analysing the impact of the most recent legal developments in smoking prevention in Switzerland.

## Data Availability

The data that support the findings of this study are available on request from SM or FW. The data are not publicly available.

## Declarations

https://www.zh.ch/de/politik-staat/gesetze-beschluesse/gesetzessammlung/zhlex-ls/erlass-170_4-2007_02_12-2008_10_01-109.html). As the questionnaire did neither address the structure or function of the human body or diseases, the Swiss Human Research Act (HRA) did not apply (Chap. 1, Sect. 1, Article 2, Scope;https://www.bag.admin.ch/bag/en/home/gesetze-und-bewilligungen/gesetzgebung/gesetzgebung-mensch-gesundheit/gesetzgebung-forschung-am-menschen.html).

## Abbreviations

95% CI: 95% confidence interval
CoRoIAR: Continuous Rolling Survey of Addictive Behaviours and Related Risks
OR: Odds ratio
SHS: Swiss Health Survey
